# Structures of foot and mouth disease virus pentamers: Insight into capsid dissociation and unexpected pentamer reassociation

**DOI:** 10.1371/journal.ppat.1006607

**Published:** 2017-09-22

**Authors:** Nayab Malik, Abhay Kotecha, Sarah Gold, Amin Asfor, Jingshan Ren, Juha T. Huiskonen, Tobias J. Tuthill, Elizabeth E. Fry, David I. Stuart

**Affiliations:** 1 Division of Structural Biology, University of Oxford, Headington, Oxford, United Kingdom; 2 The Pirbright Institute, Pirbright, Surrey, United Kingdom; 3 Helsinki Institute of Life Science and Faculty of Biological and Environmental Sciences, University of Helsinki, Helsinki, Finland; 4 Diamond Light Source, Harwell Science and Innovation Campus, Didcot, United Kingdom; Rice University, UNITED STATES

## Abstract

Foot-and-mouth disease virus (FMDV) belongs to the *Aphthovirus* genus of the *Picornaviridae*, a family of small, icosahedral, non-enveloped, single-stranded RNA viruses. It is a highly infectious pathogen and is one of the biggest hindrances to the international trade of animals and animal products. FMDV capsids (which are unstable below pH6.5) release their genome into the host cell from an acidic compartment, such as that of an endosome, and in the process dissociate into pentamers. Whilst other members of the family (enteroviruses) have been visualized to form an expanded intermediate capsid with holes from which inner capsid proteins (VP4), N-termini (VP1) and RNA can be released, there has been no visualization of any such state for an aphthovirus, instead the capsid appears to simply dissociate into pentamers. Here we present the 8-Å resolution structure of isolated dissociated pentamers of FMDV, lacking VP4. We also found these pentamers to re-associate into a rigid, icosahedrally symmetric assembly, which enabled their structure to be solved at higher resolution (5.2 Å). In this assembly, the pentamers unexpectedly associate ‘inside out’, but still with their exposed hydrophobic edges buried. Stabilizing interactions occur between the HI loop of VP2 and its symmetry related partners at the icosahedral 3-fold axes, and between the BC and EF loops of VP3 with the VP2 βB-strand and the CD loop at the 2-fold axes. A relatively extensive but subtle structural rearrangement towards the periphery of the dissociated pentamer compared to that in the mature virus provides insight into the mechanism of dissociation of FMDV and the marked difference in antigenicity.

## Author summary

This is the first structure of dissociated pentamers for FMDV, and indeed for any picornavirus. We also present the first visualization of a picornavirus particle assembled from pentamers comprising VP1, 2 and 3. The formation of regular assemblies rather than random aggregates has enabled us to solve the structure to relatively high resolution using cryo-electron microscopy. Overall there are remarkably few large-scale conformational changes in the dis-assembled pentamer from its structure in the intact virion, suggesting that the mature virus pentamer is essentially a stable endpoint. However, a small rotation in VP3 abrogates the usual pentamer interface and together with the lack of VP4 drives the abnormal inside-out association of these pentameric subunits, rather than the correct capsid assembly. This change in conformation of the dis-assembled pentamer changes the antigenic surface and presumably underlies, in part, the loss of efficacy of vaccines if native particle integrity is destroyed.

## Introduction

The *Picornaviridae* are small, non-enveloped, single-stranded RNA viruses, comprising numerous genera including the *Enterovirus genus* (*e*.*g*. poliovirus, human rhinovirus, enterovirus 71), *Aphthovirus genus* (*e*.*g*. FMDV and Equine rhinitis A virus—ERAV) and *Cardiovirus genus* (e.g. mengovirus). Foot-and-mouth disease virus (FMDV) is a highly contagious virus responsible for causing serious livestock disease. Major outbreaks of FMDV, such as the one in the UK in 2001 that also affected other EU countries, serve as a reminder of the crippling economic consequences of this highly infectious pathogen [1,2].

The virus forms an icosahedral capsid from 60 copies each of the viral proteins, VP0, VP1 and VP3, organised in the form of twelve pentamers. A final maturation cleavage of VP0 occurs in the presence of RNA, to produce VP4 (the N-terminal 85 residues of VP0) and VP2. VP1–3 are surface exposed, each adopting an 8-stranded β-barrel conformation with extended N and C-termini, with VP1 surrounding the 5-fold axes of symmetry, and VP2 and VP3 alternating around the icosahedral 3-fold axes [3–5]. VP4 is internal and varies in structure and position between different picornaviruses [6]. FMDV capsids are extremely sensitive to low-pH and elevated temperature and disassemble under these conditions [7]. Empty picornavirus capsids produced recombinantly [8] or by guanidine hydrochloride treatment to inhibit RNA synthesis, generally possess VP0 since the maturation cleavage (thought to be triggered in the context of the RNA) has not occurred and are generally less stable than their mature counterparts [9,10]. In enteroviruses, VP4 and the N-terminus of VP1 are believed to be involved in membrane penetration and have been observed to exit not only from a disassembly intermediate but also to be transiently exposed on the mature capsid [11–14].

FMDV enters host cells by receptor-mediated endocytosis. In the acidic environment of the endocytic compartments the virus particle dissociates into pentamers, releasing the RNA [15–17] into the cell to produce viral progeny. However, it remains unclear how the structure of the capsid, together with the change in pH, orchestrates the translocation of the RNA genome across the lipid bilayer of an endosomal membrane into the cytoplasm. Several studies have previously shown presumed uncoating intermediates of other picornaviruses [11,13,14,18–22], but to our knowledge there have been no studies thus far showing an intermediate particle from the FMDV infection pathway. Neither has there been any picornavirus particle reported to have formed from pentamers comprising VP1-3, although such a particle may exist for another member of the *aphthovirus* genus, ERAV [19]. Here we have determined the low-resolution structure of isolated pentamers formed by the acid treatment of infectious, native FMDV particles and in addition have captured an icosahedral assembly of these pentamers, which have associated with the reverse curvature to their normal organisation in the mature native particle. These ‘inside-out’ particles were characterised at a resolution of 5.2 Å, using cryo-electron microscopy (cryo-EM). We find that the structures of ordered portions of both isolated and assembled pentamers are generally very similar not only to each other but also to the corresponding regions of the mature capsid although a small rotation of VP3 has occurred on dissociation of the pentamer.

## Methods

### Growth and purification of virus

FMDV serotype A10_61_ was grown in the baby hamster kidney cell line, BHK-21 (BHK-21 cells (clone 13) have been maintained in the Pirbright Institute since 1965) and purified as previously described [23]. Briefly, infected cells were harvested at maximum CPE, freeze-thawed and lysates concentrated by precipitation with ammonium sulphate. Particles were pelleted through a cushion of 30% (w/v) sucrose in PBS and purified by sedimentation in a 15–45% (w/v) sucrose density gradient in PBS. Purified virus was pooled from peak gradient fractions, quantified by absorbance at 260nm (where an optical density of 7.7 = 1 mg/ml) and stored at -80°C.

### Low pH-induced dissociation of virus

Samples of purified virus prepared as described above (in PBS containing approximately 30% sucrose) were adjusted to pH 6.3 by 10-fold dilution in 107 mM NaCl, 50 mM citric acid:sodium phosphate, 0.1mg/ml BSA, 2mM CaCl_2_ pH 6.3 for 2 minutes at room temperature to promote capsid dissociation, then neutralised by addition of HEPES pH 8.0 to 166 mM. Control samples were treated in parallel by dilution with PBS. The first low pH-treated and control samples were used to assess capsid integrity by sedimentation through 15–30% sucrose gradients at 28,000 rpm in an SW32 rotor (approximately 141,000 x g) for 4 h at 4°C. Gradients were fractionated from the top and capsid material in the fractions adsorbed onto 96-well plates and detected by indirect ELISA using polyclonal antibody against serotype A FMDV and an HRP-conjugated secondary antibody.

### Inactivation and innocuity testing

Residual infectivity in acid-treated samples was inactivated by the addition of binary ethylenimine (BEI) to a final concentration of 1 mM and shaking at 37°C for 24 h [24]. A proportion (5%) of the inactivated sample was removed for innocuity testing, the remainder of the sample was treated with a second round of inactivation and stored sealed and frozen at -80°C until transported out of the Pirbright high containment area for analysis by electron microscopy. Innocuity testing was carried out by the World Reference Laboratory for foot-and-mouth disease (WRLFMD) at The Pirbright Institute. Briefly, the sample was exposed to cultured primary calf thyroid cells (BTY) for 3 days, cells were freeze/thawed and lysates exposed to new BTY cells for a further 3 days. All cultures were observed daily to confirm the absence of virus induced cytopathic effect.

### Preparation of samples for electron microscopy

Initial samples were taken from the sucrose gradient purification described above. In subsequent preparations the gradient preparation step was omitted and acid treated samples were simply concentrated. In both cases samples were desalted using 7K MWCO 5ml Zeba Spin Desalting columns (Thermo Scientific) and concentrated approximately 40-fold using 0.5ml 100kDa MWCO Amicon concentrators (Merck Millipore Ltd.) by spinning at 5000*g* to reduce the volume to 50μL.

### Negative stain electron microscopy

3μL drops of sample from before and after concentration were applied to glow-discharged copper grids (Formvar/carbon-coated grids Electron Microscopy Sciences) and allowed to adsorb for 60s. After blotting away the excess sample, the grids were washed twice with deionized water and then stained with 2% (w/v) Uranyl Acetate for 60s, after which the excess stain was blotted away. Imaging was performed on a 120kV Tecnai T12 transmission cryo-electron microscope. Data were acquired on a 4k x 4k FEI Eagle camera at a magnification of 67000x, corresponding to a pixel size of 1.68 Å.

### Cryo electron microscopy data collection

4μL drops of concentrated sample were applied to glow discharged holey carbon-coated copper grids (C-flat, CF-2/1-2C, Protochips) in a humidity-controlled chamber, at 90% humidity using a Vitrobot (FEI, Hillsboro, Oregon). After 30s incubation the grids were blotted for 3s before being plunged into liquid ethane cooled in liquid nitrogen.

Data were collected using a direct electron detector (K2 Summit, Gatan, Abingdon) on a 300kV Tecnai F30 Polara electron microscope (FEI) with an energy filter (GIF Quantum, Gatan, Abingdon) in zero-loss mode (0–20 eV energy slit). All movies had 25 frames, 0.2s each, and were recorded 1.5–3.5μm underfocus in single electron counting mode, at a dose of 25 electrons/Å^2^ and a nominal magnification of 37,037x (pixel size 1.35 Å), using SerialEM.

### Structure determination

All the frames of the movies, except the first two, were averaged to produce motion corrected micrographs (MOTIONCORR [25]). The first two frames were excluded to reduce the effects of initial drift. Astigmatic or micrographs with a significant drift were not used for subsequent analyses. Data analysis used RELION 1.3 [26] with gold standard refinement procedures for structure determination [27,28].

The grids contained both isolated pentamers and particles. For the structure determination of the latter, particles homogenous in their structure were selected by reference-free 2D classification, followed by further triage using 3D classification. The X-ray structure of a native FMDV A10_61_ particle (coordinates 1ZBE) [29] was low-pass filtered to 50 Å resolution and used as an initial template for 3D classification[26]. This step was then repeated using the 3D class that looked the most different from a native particle as a template. A total of 8,077 particles were used from 1570 micrographs for the final structure determination to 5.19 Å, at a Fourier shell correlation cut-off of 0.143 (so-called ‘gold standard’ procedure, S1A Fig). At all stages icosahedral symmetry was enforced.

The structure of the isolated pentamers was determined starting from a pentamer of the native FMDV A10_61_ virus coordinates rigid body fitted into the map using Chimera. The density corresponding to the pentamer was cut out using ‘model as a mask’ in Chimera. Most of the pentamer particles picked were side-views, since (assuming these are randomly arranged about the 5-fold axis) these contribute to more even coverage in Fourier space for structure determination than face views. 4,260 pentamer particles from 1,500 micrographs contributed to the final structure determination of the standalone pentamer to 8.2 Å, at the FSC cut-off of 0.143 (S1B Fig). C5 symmetry was enforced on the reconstruction of the isolated pentamer.

The local resolution distribution of both maps was calculated using RESMAP (http://resmap.sourceforge.net/) (S2 and S3 Figs).

To obtain the particle structure, *Coot* [30] was used to rigid-body fit manually an initial model obtained from the native A10 crystal structure into the EM density. The structure was further adjusted and refined manually and then using *phenix*.*real_space_refine* [31] iteratively refined in real space until the refinement statistics were satisfactory. The final fitting of the model into the whole cryo-EM map had a cross-correlation (CC) of 0.77, 0.81 in the region of the atoms (within 2 Å of an atom), and showed no rotamer outliers (S4 Fig). The structure of one pentamer from the particle was extracted and rigid body fitted into the map of the isolated pentamer (using Chimera), to yield CC = 0.88 for the whole pentamer.

### PISA analysis

The coordinates for two neighbouring pentamers were cut out from the model of the entire assembly and saved as separate PDB files for use in the PISA server [32] to analyse the inter-pentamer interface interactions.

## Results

### Determination of the structure of a dissociated capsid pentamer and a novel FMDV assembly

**Fig 1:**
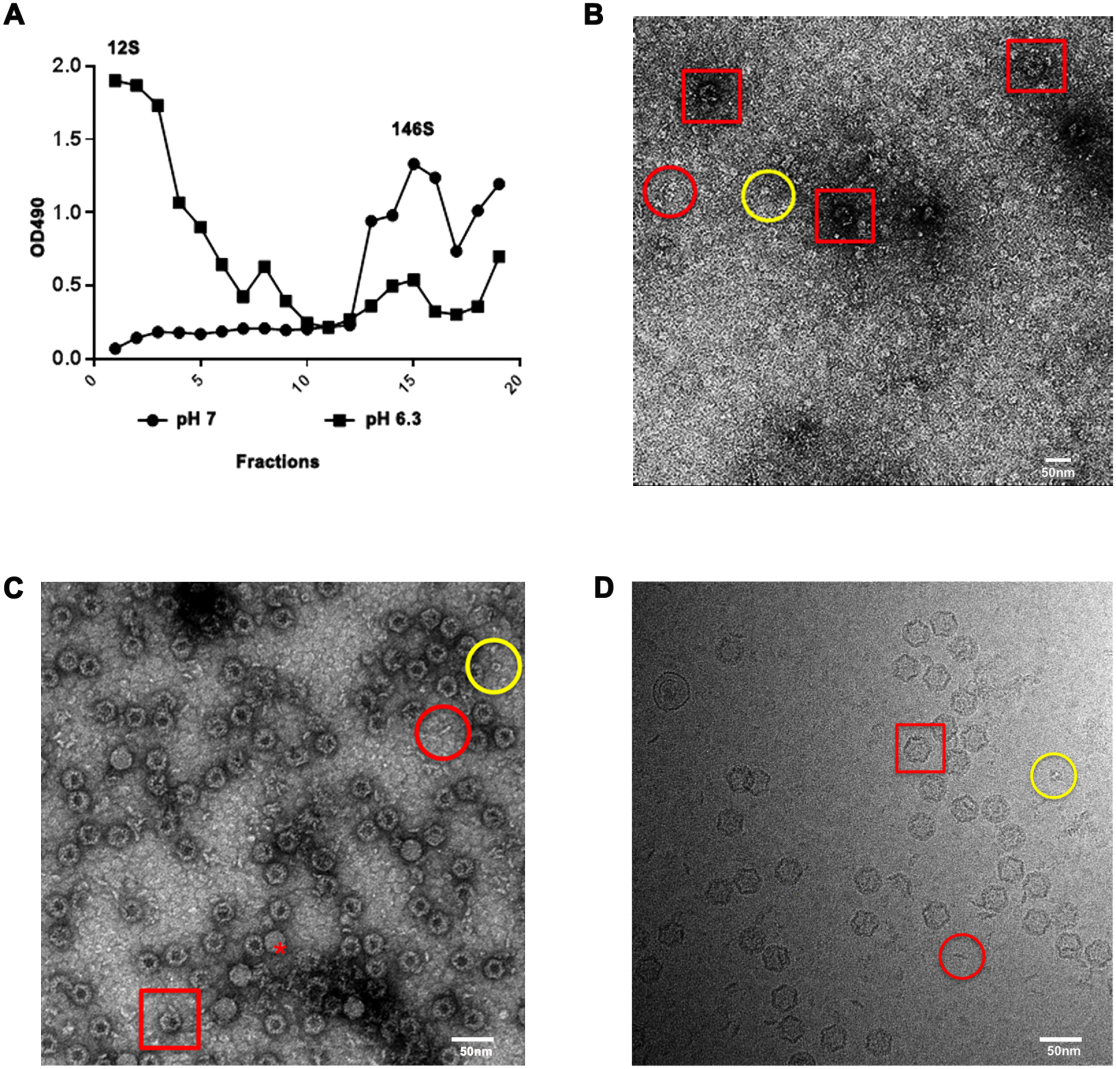
Analysis of the inside-out FMDV particles. (A) Sucrose gradient analysis of particles before and after dissociation to pentamers. (B) Negative stain EM analysis of dissociated particles before concentration and (C) after 40-fold concentration. (D) Cryo-electron microscopy images of low-pH treated FMDV capsids. Red square: empty inside-out particles with stain inside. Red circle: pentamer, side view. Yellow circle: pentamer, top view and Red asterisk: a small proportion of residual native particles.

Native FMDV particles were subjected to low pH treatment for two minutes, before the pH was restored to neutral (Methods). The status of viral particles in low pH and mock treated samples was assessed by analysing their sedimentation during ultracentrifugation through sucrose gradients. The majority of capsid material in low pH treated samples sedimented at the rate expected for dissociated pentamers and the majority of capsid material in mock treated samples sedimented at the rate expected for intact virus (Fig 1A). This confirmed that these conditions specifically induced the expected dissociation of the majority of virus particles into capsid pentamers. Samples of low-pH dissociated pentamers were inactivated by treatment with binary ethylenimine. Initial data were derived from density gradient purified material, desalted and concentrated, subsequent preps used just desalting and concentration (Methods). No significant differences in either negative stain or cryo-EM images were seen between the samples. Initial screening of these samples using negative-stain EM before concentration, showed the presence of very few particles and with the majority of visible material having an appearance consistent with pentamers (Fig 1B). The same analysis of material after 40-fold concentration revealed an unexpectedly large number of particles with a previously unobserved morphology (Fig 1C). This material was also prepared for data collection using high-end cryo-EM and the raw images obtained showed the presence of these particles, a very few full particles and a high concentration of dissociated pentamers (Fig 1B, 1C and 1D). The pentamers had preferred orientations corresponding to ‘top-views’ on continuous carbon used for negative stained EM (Fig 1B and 1C), and edge views in vitreous ice (Fig 1D). The dissociated pentamers appeared to be hydrophobic, since they stuck to the carbon support of the cryo-EM grids or aggregated. Only a small fraction of the separated pentamers dispersed in the vitreous ice, making it challenging to solve a high-resolution structure of a standalone pentamer. Due to the limitation of having only a few thousand particles (the majority of which were side views) this structure was determined only to low resolution (8 Å). The side views provided a reasonable sampling in reciprocal space, as shown in S5 Fig.

Analysis of the unusual assemblies revealed them to be slightly expanded compared to native FMDV capsids (312 Å in diameter compared to 297 Å). The particles were empty, as shown by the black spots of heavy-metal stain inside them and they had slightly concave faces as if the outer shell had been sucked inwards.

**Fig 2:**
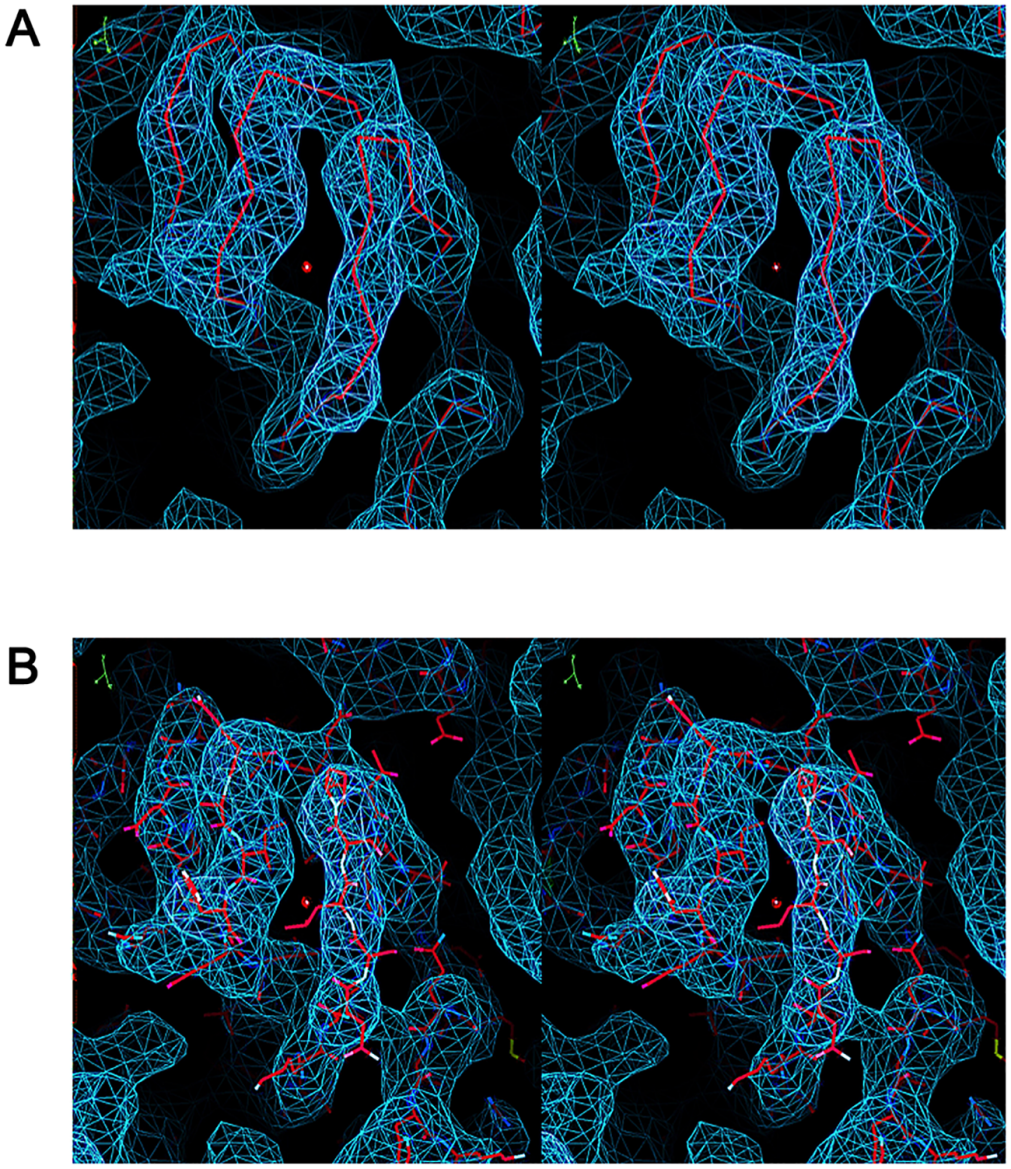
Atomic model fitting of the inside-out particle into the EM density. The 5.2 Å electron density map (blue mesh) of the inside-out particle allowed the unambiguous fitting and refinement of the PDB (1ZBE) model. Stereo-views of the same region with the (A) C-alpha backbone and the (B) atomic representation of the model fitted into the EM density are shown in red.

**Fig 3:**
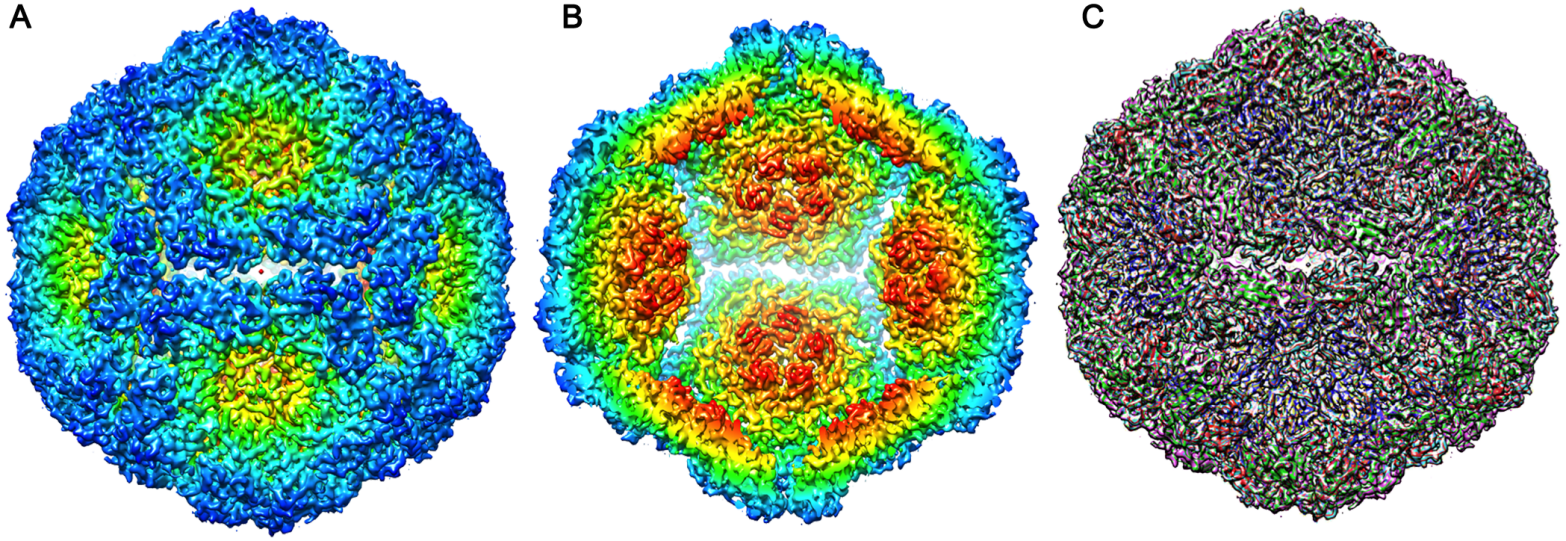
5.2 Å structure of the FMDV inside-out particle. (A and B) show the particle and a cross-section through the particle viewed down the two-fold icosahedral symmetry axis, coloured radially from the centre (<110 Å: red; 120–140 Å: yellow; >150 Å: blue). (C) Atomic fitting of the structure into the electron density map (transparent grey render) to generate the whole virus structure of the inside-out particle. Individual protein chains are coloured blue: VP1, green: VP2 and red: VP3.

From a total of 1,570 micrographs, some 8,077 particles contributed to the final structure determination to 5.2 Å for the empty shells, at a Fourier shell correlation cut-off of 0.143 (S1A Fig). For the isolated pentamers 4,260 particles led to a reconstruction at a final resolution of 8.2 Å using the same criterion (S1B Fig). The resolution of the unusual particles was sufficient to allow fitting of the native FMDV serotype A10_61_ coordinates (PDB 1ZBE) into the density (Fig 2). Convincing initial rigid-body fitting, assuming that the shell represented an expanded particle with collapsed 5-folds, was possible only when the whole pentamer of the native virus was turned upside down and VP4 removed. It was then immediately apparent that the structure of the pentamer seen in the mature virus was broadly maintained, with VP4 not detected and portions of the N-termini of VP1 and VP2 disordered. Thus an inverted FMDV A10 pentamer (PDB 1ZBE) could be fitted into the electron density with a CC of 81% around the atoms. The particle clearly represents a re-assembly product of pentamers and, since it is radically different to the normal assembly product for the virus we refer to it below as an inside-out particle. From the initial pentamer fitting, the structures of VP1-3 were rebuilt using *Coot* and refined with *phenix*.*real_space_refine* to give a generally reliable atomic model (see Methods). The final model comprises residues 27–208 of VP1, 29–210 of VP2 and 1–221 of VP3 (Fig 3).

### The inside-out particle comprises relaxed pentamers after disassembly

**Fig 4:**
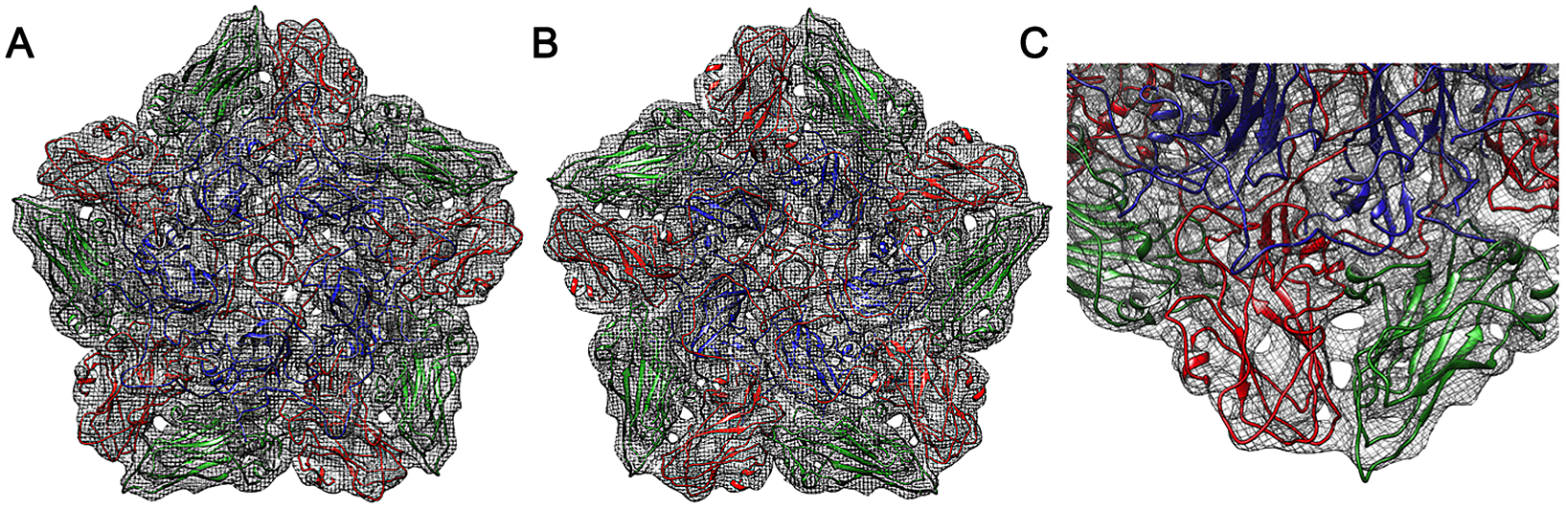
Atomic model fitting of the inside-out particle pentamer structure into the isolated pentamer density. The isolated pentamer density (grey mesh) is observed down the icosahedral 5-fold axis from the (A) inside and (B) outside (relative to the inside-out pentamer) showing how the VP1, VP2 and VP3 (cartoon representation coloured as in Fig 3) are arranged in a dissociated pentamer. (C) A zoomed-in view to show the quality of the fitting.

The density for the isolated pentamer correlated extremely well with density for a pentamer cut out from the inside-out shell (CC = 0.88), and at the resolution of the analysis no structural rearrangements were obvious (Fig 4). In contrast density derived from the coordinates of the native virion (PDB 1ZBE) agreed less well (CC = 0.81), see S6 Fig, due to some subtle rearrangements in the structure (see below). This confirms that the inside-out assembly described above comprises dissociated pentamers in their post-uncoating, relaxed state. In neither of the reconstructions was density observed for VP4, so it was omitted from the native PDB coordinate fitting. Furthermore, no density was observed for the N-terminal VP2 hairpin loop of the native structure. We have used the structure of the pentamer from the inside-out icosahedral assembly as a model for the isolated pentamer in the remainder of the paper, since they are indistinguishable.

### Key interactions that hold the inside-out particles together

**Fig 5:**
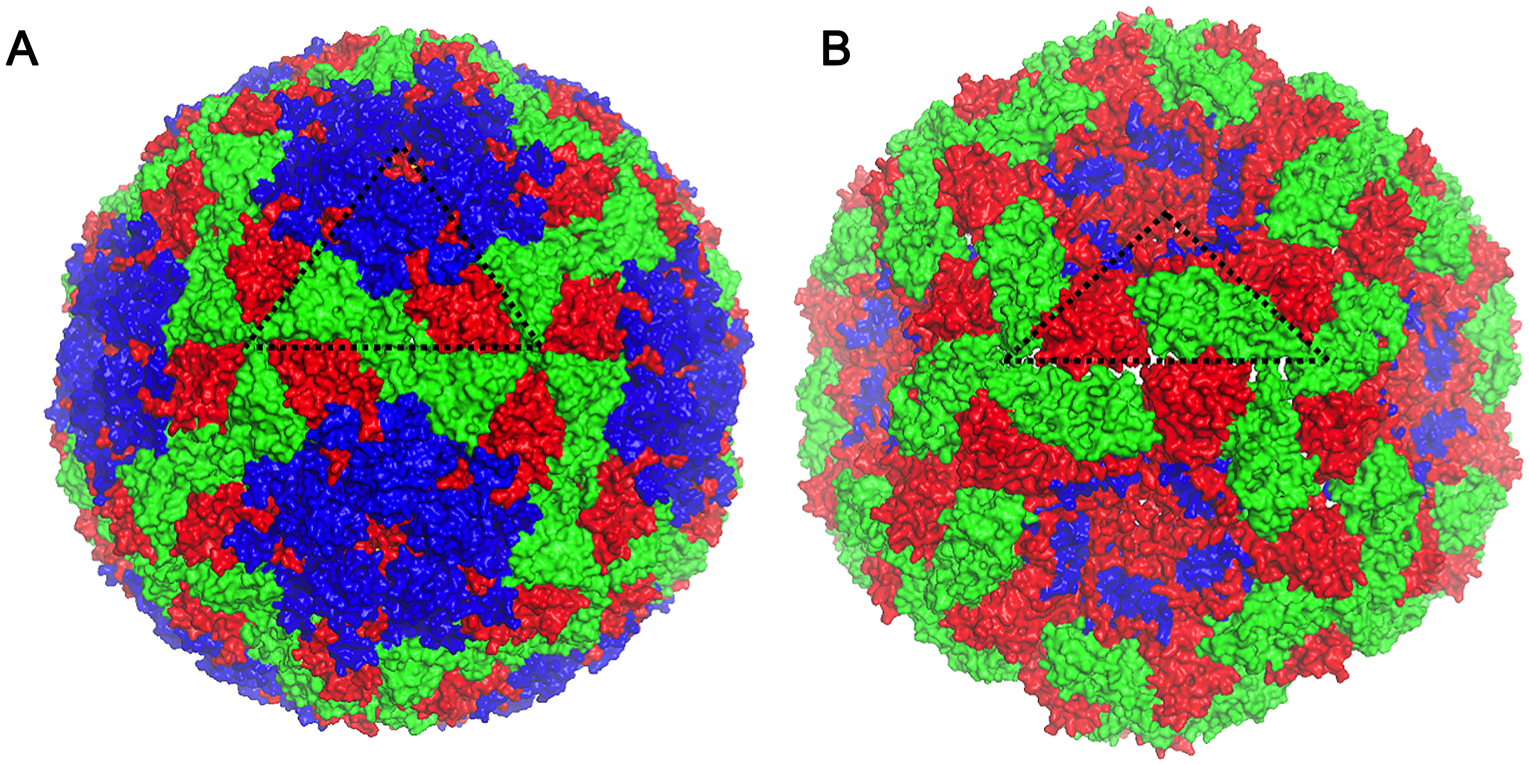
A comparison of the native FMDV capsid structure to that of the inside-out particle. A comparison of surface representations of the (A) native crystal structure (1ZBE) and the (B) inside-out particle viewed down the 2-fold icosahedral symmetry axis. A protomer comprising of VP1 (blue), VP2 (green) and VP3 (red) is highlighted by a black triangle. A gap at the two-fold axis, and the reverse orientation of VP2 and VP3 can be seen in the inside-out particle.

**Fig 6:**
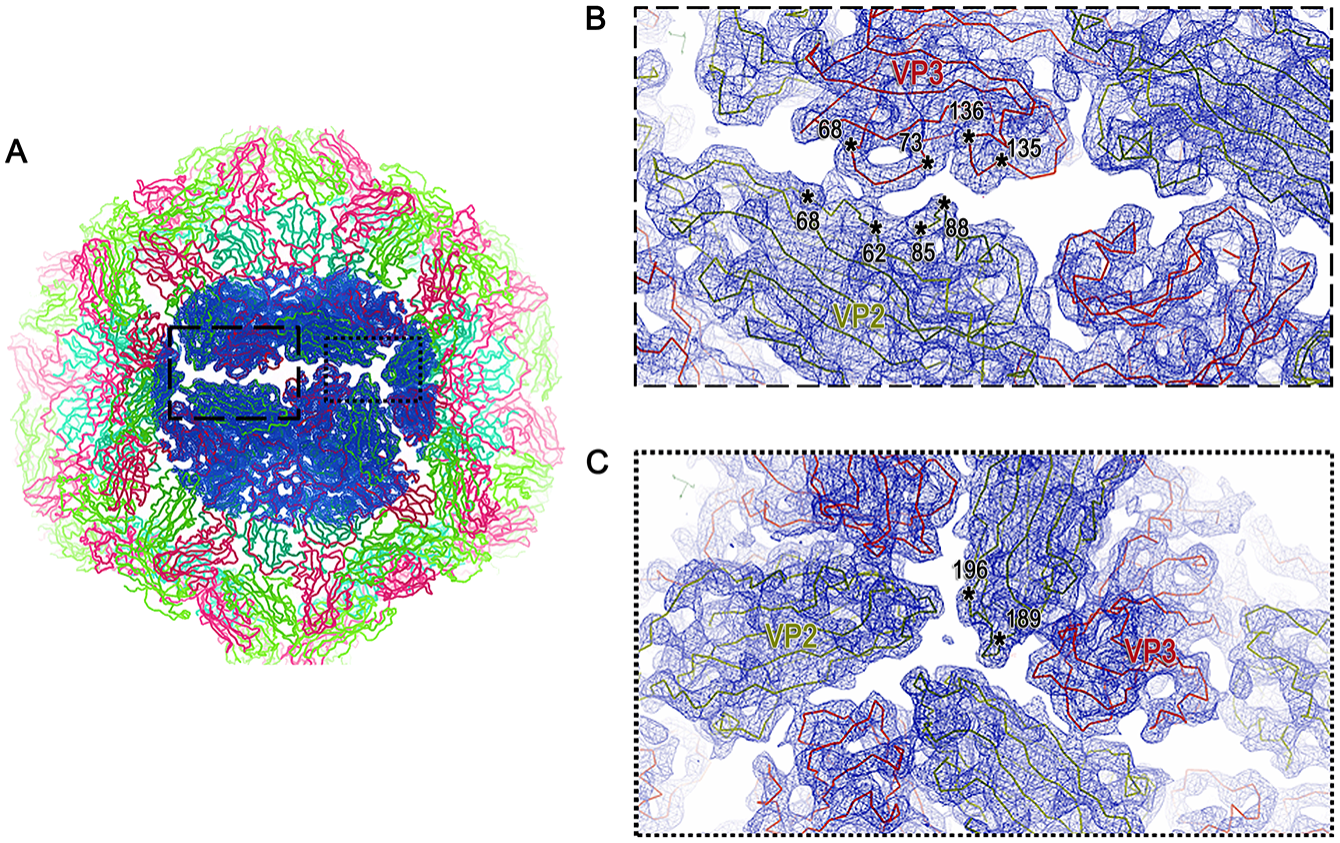
Stabilising interactions at the inter-pentameric interfaces of the inside-out particle. Interface analysis of the inside-out particle. (A) Black rectangles high-light the interacting regions between the 2- and 3-fold icosahedral symmetry axes and around the 3-fold symmetry axis with the proteins drawn as ribbons coloured as in Fig 3. (B) Blue mesh electron density with the VP2 (green) and VP3 (red) loops shown interacting at the two-fold axis with residues involved labelled. (C) The symmetry-related copies of VP2 and VP3 from three pentamers in close proximity at the three-fold axis, The VP2 residues might also form capsid-stabilising interactions.

The interactions at the pentamer interfaces in the inside-out particle derived by analysis of the EM density at the protein interface of VP2-VP3 (Fig 5), showed that the pentamer-pentamer interactions are weak and have fewer hydrogen bonds and salt bridges than observed in the native capsid [29]. The interacting surface area [32] is dramatically reduced (S1 Table), but some key interaction patches hold the inside-out particle together (Fig 6). These lie at the mid-point between the 2- and 3-fold axes (Fig 6B). The BC loop of VP3 (residues 68–73) and the adjacent EF loop (residues 135–136) interact with a strand of VP2 composed of residues 62–68 and the CD loop (residues VP2 85–88) respectively, of the opposing pentamer to stabilise the assembly. Although it is hard to say with certainty at a resolution of ~5 Å, there might be stacking hydrophobic interactions between VP3 Arg72 and VP2 His88. VP2 Lys63 is also likely to hydrogen bond with the main chain of VP3 Asp70 or VP3 Thr71. In addition the 3-fold axis shows the symmetry related copies of VP2 from three pentamers in fairly close proximity to each other. While the closest Cαs of VP2 are more than 8 Å apart, there may be some van der Waals interactions between the side chains (Fig 6C).

### Dissociated pentamers differ significantly from those comprising the mature capsid

There are a large number of significant structural changes between the dissociated pentamers and those in the mature virion (S6 Fig). Unsurprisingly many of these are on the surface that is internal in the mature virus but external in the inside-out particle. As noted above, the N-termini of VP1 and VP2 in the inside-out particle are disordered (the initial 26 and 28 residues respectively are missing,). Both of these regions are involved in interactions around the edge of the pentamer, which contribute to maintaining the coherence of the native viral capsid, but these interactions are lost in the pentamer-pentamer interactions of the inside-out particle. The loss of VP4, along with the disorder of the VP1 N-terminus and VP2 N-terminal hairpin loop (residues 1–28) relax the structure perhaps allowing the translation in the VP2 β-sheets of between 3–6 Å, part of a generally looser packing of the proteins in the inside out assembly (S7 Fig). As noted above, compared to the mature virus particle, pentamer-pentamer interactions are tenuous (PISA analysis, S1 Table) and the pentamers are loosely packed, so that the polypeptide of the two-fold axes helices that abut in the mature virus are separated by a gap of between 20 Å and 9 Å (the side chains narrow this to 13 Å and 3 Å respectively (Fig 5B) [8].

It is interesting to note that the loss of VP4 and disordering of the N-terminal region of VP1 in the relaxed pentamers recapitulate what has been seen in empty picornavirus particles, where modified enterovirus particles that have lost RNA and VP4 [11,13,20–22] have been observed that are generally considered models of uncoating intermediates.

**Fig 7:**
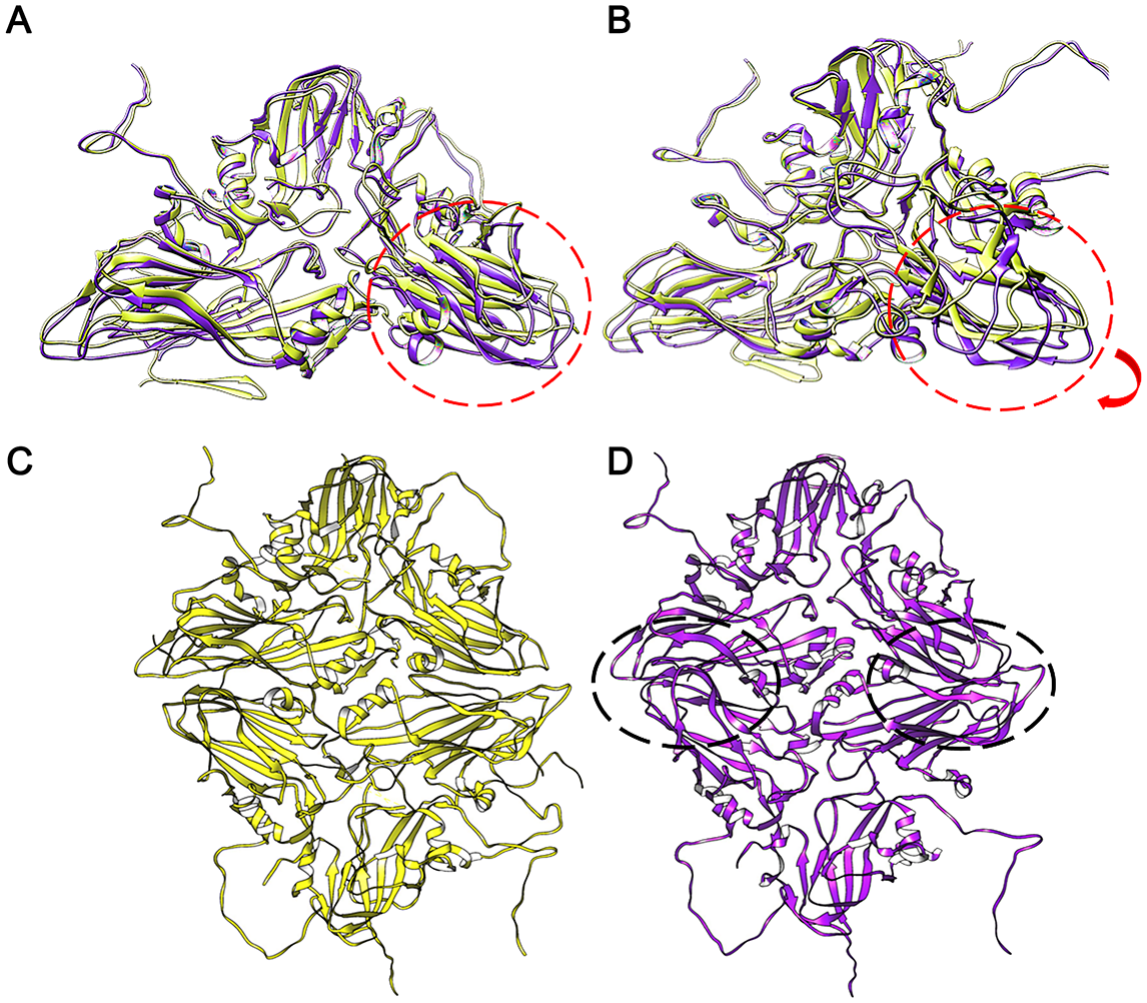
Conformational changes highlighting the differences between the native FMDV capsid and the inside-out particle. Depth-cued structures, after VP1 superposition, of native (yellow cartoon) and inside-out particle protomers (purple cartoon), highlighting the conformational changes in VP3 (red circles). (A) The shift in VP3 beta-sheets in the inside-out particle is highlighted by red circles. (B) The 20° y-axis rotated view of the protomers emphasises the 4.6° rotational shift in VP3 (red arrow). (D) The structural clashes which would occur between the purple protomers at the VP2-VP3 two-fold axis, had they assembled in the same orientation as the native, are highlighted by the black circles, and a (C) native arrangement of protomers is shown for comparison.

There are additional, unexpected, conformational rearrangements elsewhere in the pentamer. Most notable is a 4.6° rotation of the bulk of VP3 compared to its position in the native virion, when the protomer structures are superimposed on VP1 (Fig 7). When the two VP3 molecules are superimposed, the Cαs overlap with an RMSD of 2Å (SHP [33]), showing that the structure largely moves as a rigid body, however some loops (EF, HI, DE, FG) have shifted in the range of 4–9 Å, the largest conformational changes seen in the protomer (Fig 7).

## Discussion

How the RNA genome exits picornaviruses remains poorly understood, but it is assumed that some structural changes to the mature capsid allow RNA to exit the capsid and be translocated to the cytoplasm of the host cell. Whilst in some picornaviruses (e.g. enteroviruses [11–13,20–22]) putative disassembly intermediates have been visualised, in FMDV this is not the case, thus any such disassembly intermediate between the mature capsid and the end-product of dissociated pentamers is likely to be transient and difficult to capture. Here we have determined the structure of dissociated pentamers and have also shown that *in vitro* these can assemble into icosahedral structures of roughly similar sizes to mature virus, but radically different in their organisation (being inside-out). It is significant that VP4 was absent in these particles, consistent with them being formed from dissociated pentamers from mature capsids. Indeed Burroughs *et al* [34] showed that VP4 is absent from sucrose density purified pentamers and our initial cryo-EM data on the reassembled particles was derived from material purified in exactly this way. This finding was confirmed by the higher correlation seen between our fitted inside-out pentamer structure and the density of the standalone pentamer, than between that of the native pentamer with this density. Thus correct re-assembly of pentamers is hindered by the absence of both VP4 and the N-terminus of VP1 and the accompanying minor structural changes that have occurred in VP2 and in particular the rotation of VP3, which would introduce steric clashes with the opposing VP2 in the native particle structure (Fig 7D). Since we are looking at an end-product it is impossible to know whether the rotation in VP3 occurred prior to uncoating, perhaps triggering particle dissociation, or after the uncoating had already taken place. Undoubtedly the missing regions of the capsid would be involved in RNA interactions, which facilitate the correct particle assembly. The inside-out particles we observe are unlikely to be biologically relevant but the pentamers from which they are formed are.

The only other significantly different type of particle structure reported for a picornavirus is that for another member of the *aphthovirus* genus, ERAV [19] where a more expanded particle was visualized (for a sample which had been stored for some time) with the pentamers tenuously associated. This 20-Å resolution structure was mooted to be a disassembly intermediate, however taking our data into consideration it seems equally likely that this was also the product of re-assembled dissociated pentamers. In contrast a low pH structure of ERAV, thought to represent a pre-dissociation intermediate only shows significant changes on the inside of the particle [18].

The differences in the structure of the disassembled pentamer include the rearrangement of polypeptide chains, which will inevitably alter the antigenic properties [35], contributing to the ineffectiveness of vaccines stored for long periods or subjected to adverse conditions, where the integrity of the virus capsids will be seriously compromised. This structural information may be used to help engineer more stable native particles for vaccine formulation.

## Supporting information

S1 Table PISA interface statistics. The VP2-VP3 interface has been analysed using PISA for both the native and inside-out A10_61_ structures.
(DOCX)

S1 Fig Resolution determination at FSC cut-off of 0.143. (A) Resolution determination of the inside-out particle at 5.2 Å and of (B) the isolated pentamer at 8.2 Å at the gold-standard Fourier Shell Correlation (FSC) cut-off of 0.143.
(TIF)

S2 Fig Resmap local resolution determination of the inside-out particle. Local resolution determination of the FMDV inside-out particle, using Resmap. (A) The full particle and (B) a cut open view.
(TIF)

S3 Fig Resmap local resolution determination of the isolated pentamer. The local resolution map, generated using Resmap, of the isolated pentamer from both sides.
(TIF)

S4 Fig Final refinement statistics of the inside-out particle. (A) The final refinement statistics of the inside-out particle and (B) the associated Ramachandran plot.
(TIF)

S5 Fig Angular sampling of the pentamer structure. The angular sampling frequency (shown by the length of the rod) and distribution (shown by the rod direction) of particles of the isolated pentamer used to produce the 8.2 Å structure, drawn in Chimera.
(TIF)

S6 Fig Comparison of the fitting of the inside-out pentamer structure (VP1 (blue), VP2 (green) and VP3 (red)), to that of the native pentamer (yellow) into the electron density map of the isolated pentamer (grey mesh). Two different views of the two pentamers shown to highlight the difference in the structures that allows the better fitting of the atomic model from the inside-out particle to fit into the electron density for the isolated pentamer. The view on the left is looking down on the pentamer, as if from outside the native virus, whilst the view to the right is almost edge-on with the outer surface in the native virion facing upwards. The missing density for the VP2 hairpin loop of the native structure and the better fitting of the VP3 (red) β-sheets from the inside-out particle model are visible (black arrows point to these structures).
(TIF)

S7 Fig Two different views showing the relaxed VP2 β-sheets of the inside-out particle (green) in comparison to ones from the native capsid (purple). VP4 (yellow), the N-terminus of VP1 (dark blue in the background) in the case of the native capsid and VP3 (red) can also be seen.
(TIFF)
