# Supplementary material for: Structures of foot and mouth disease virus pentamers: Insight into capsid dissociation and unexpected pentamer reassociation
Source: PLoS Pathog. 2017 Sep 22;13(9):e1006607. doi: 10.1371/journal.ppat.1006607 (PMC5656323; doi:10.1371/journal.ppat.1006607)
Supplement: S1 Table — The VP2-VP3 interface has been analysed using PISA for both the native and inside-out A1061 structures. (DOCX) [file ppat.1006607.s001.docx]

**S1 Table**

| **VP2-VP3 interface** | **Interface area (Å^2^)** | **No. of H-bonds** | **No. of salt bridges** |
| --- | --- | --- | --- |
| **Native A10_61_ (1ZBE)** | 2772.9 | 38 | 6 |
| **Inside-out A10_61_** | 927.0 | 8 | 0 |
