# Supplementary figures and images for: Structures of foot and mouth disease virus pentamers: Insight into capsid dissociation and unexpected pentamer reassociation
Source: PLoS Pathog. 2017 Sep 22;13(9):e1006607. doi: 10.1371/journal.ppat.1006607 (PMC5656323; doi:10.1371/journal.ppat.1006607)

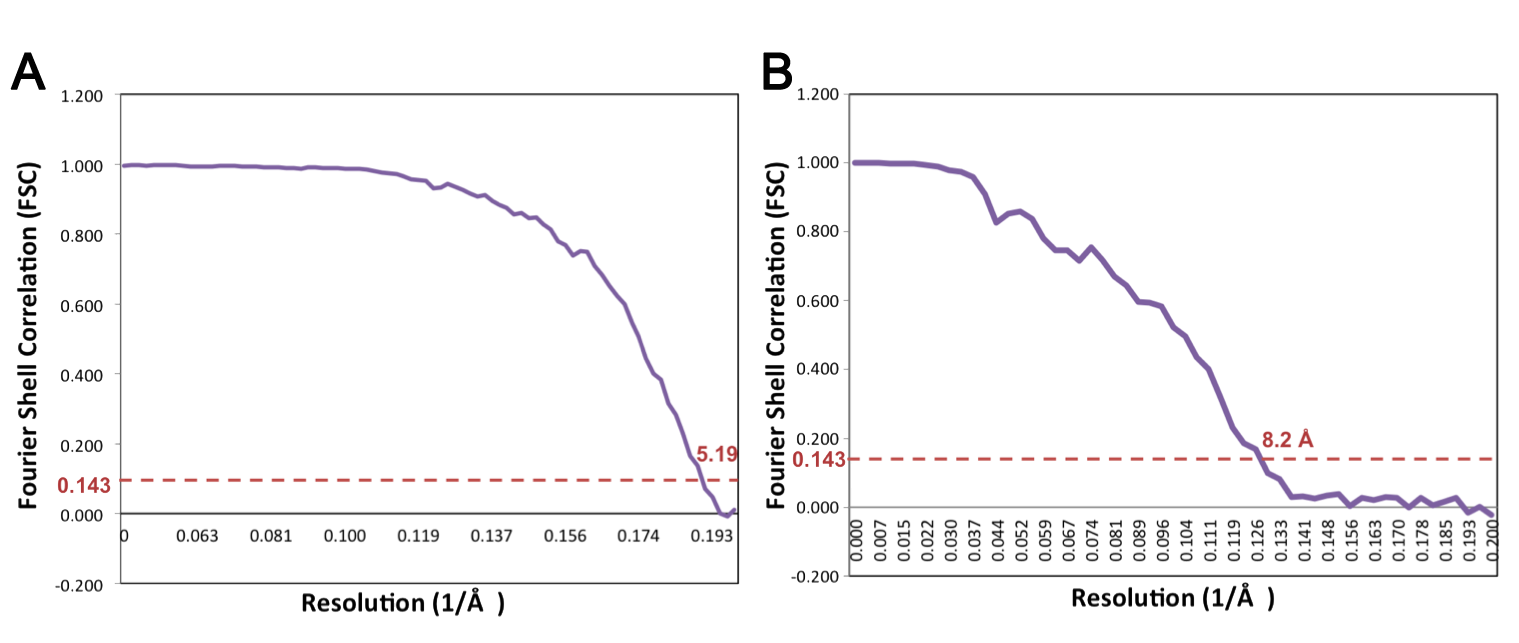

Supplement: S1 Fig — (A) Resolution determination of the inside-out particle at 5.2 Å and of (B) the isolated pentamer at 8.2 Å at the gold-standard Fourier Shell Correlation (FSC) cut-off of 0.143. (TIF) [file ppat.1006607.s002.tif]

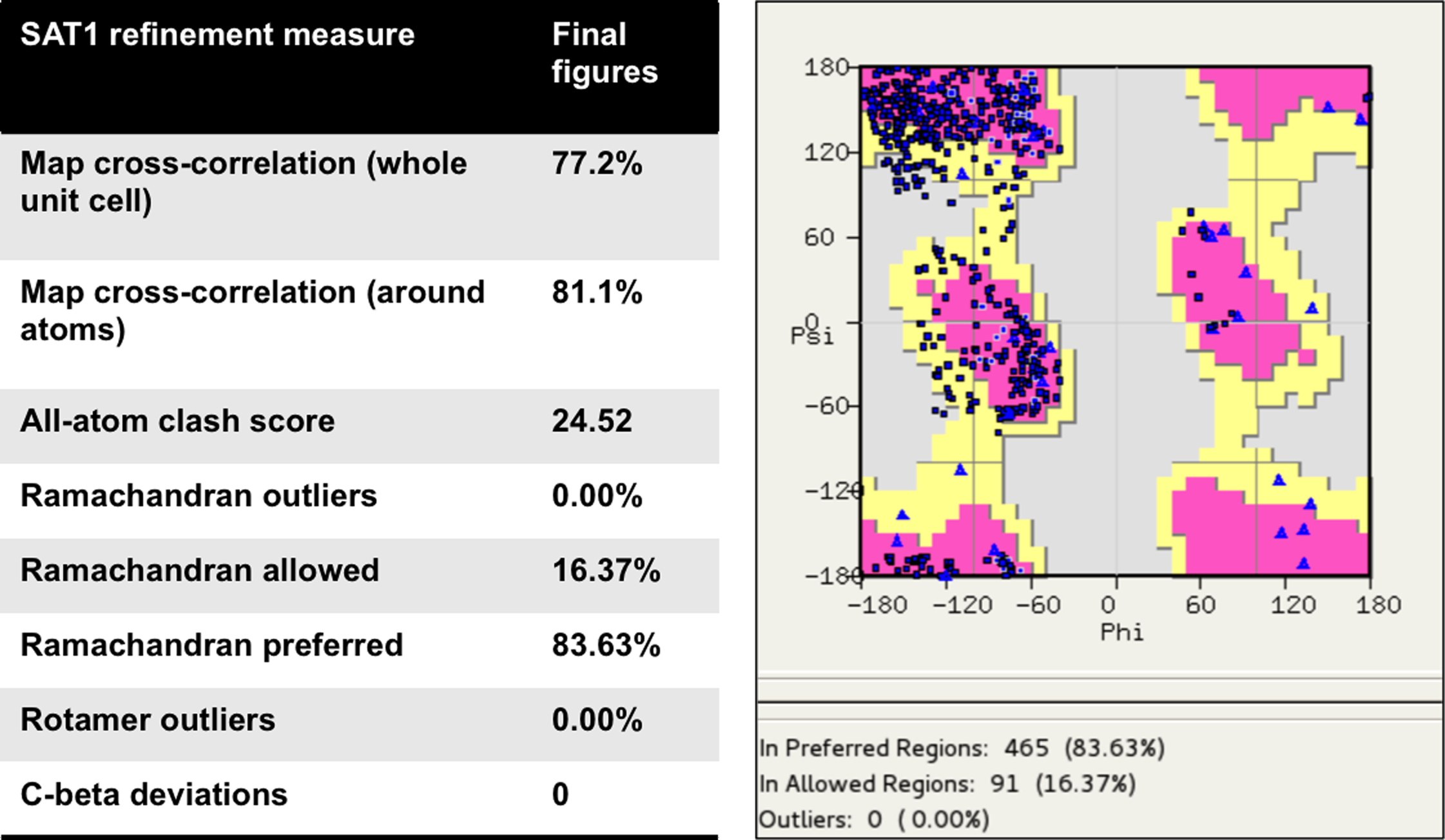

Supplement: S2 Fig — Local resolution determination of the FMDV inside-out particle, using Resmap. (A) The full particle and (B) a cut open view. (TIF) [file ppat.1006607.s003.tif]

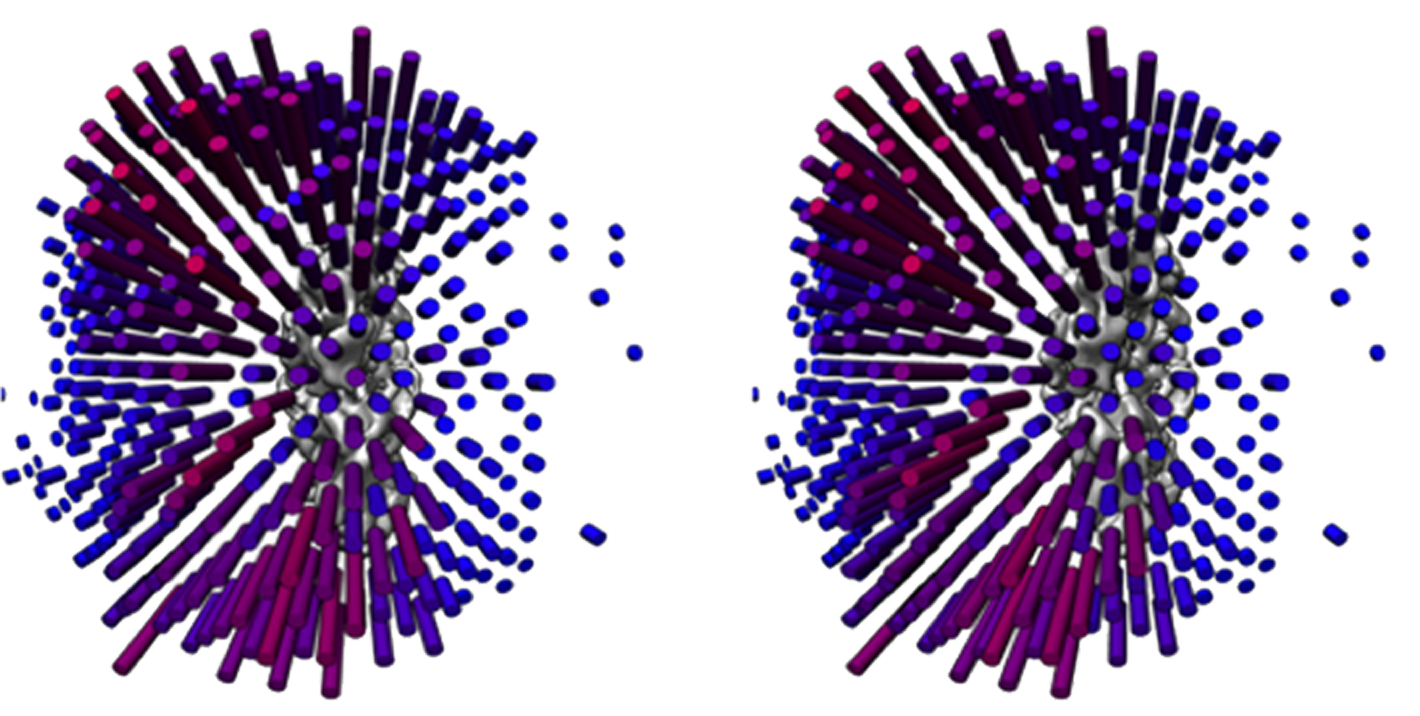

Supplement: S3 Fig — The local resolution map, generated using Resmap, of the isolated pentamer from both sides. (TIF) [file ppat.1006607.s004.tif]

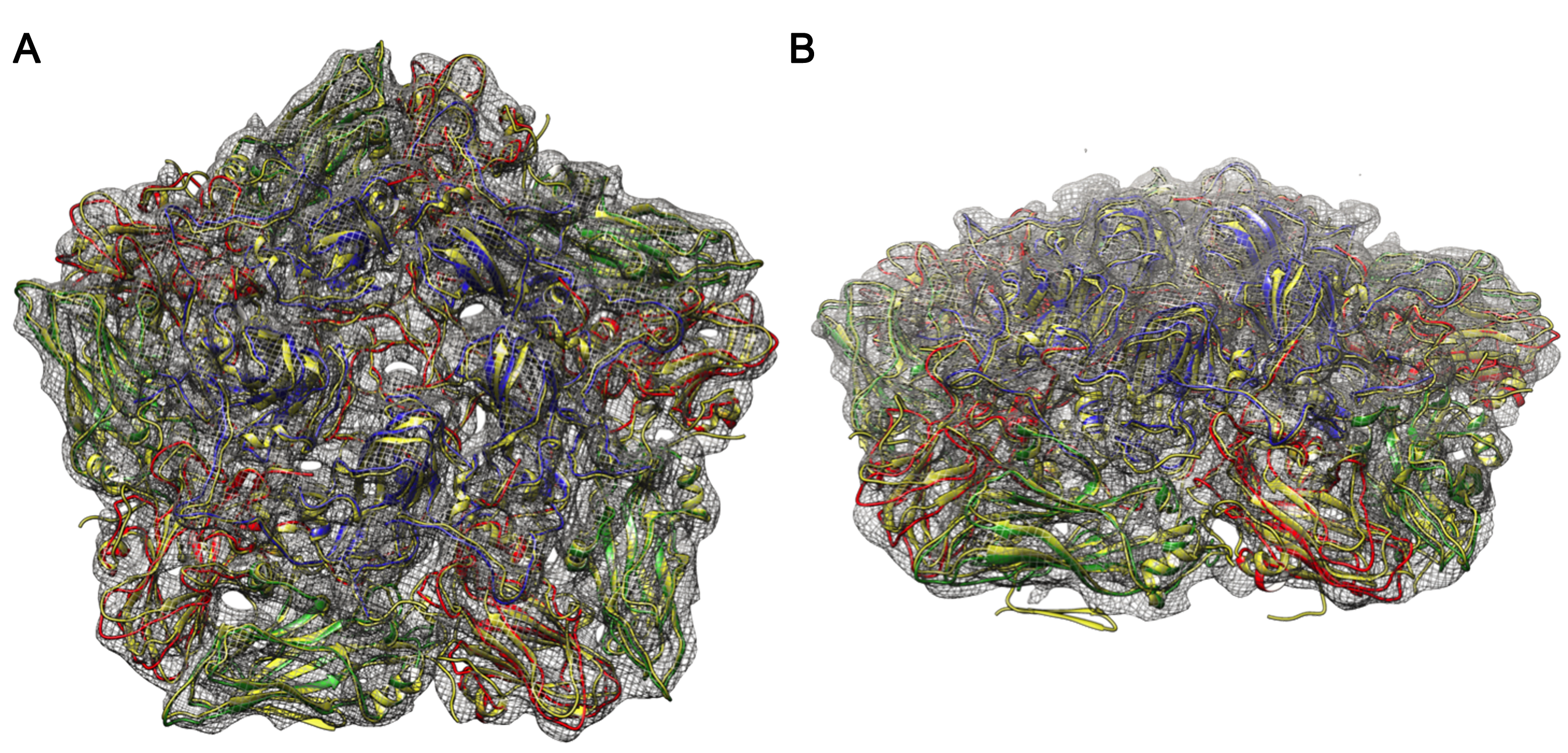

Supplement: S4 Fig — (A) The final refinement statistics of the inside-out particle and (B) the associated Ramachandran plot. (TIF) [file ppat.1006607.s005.tif]

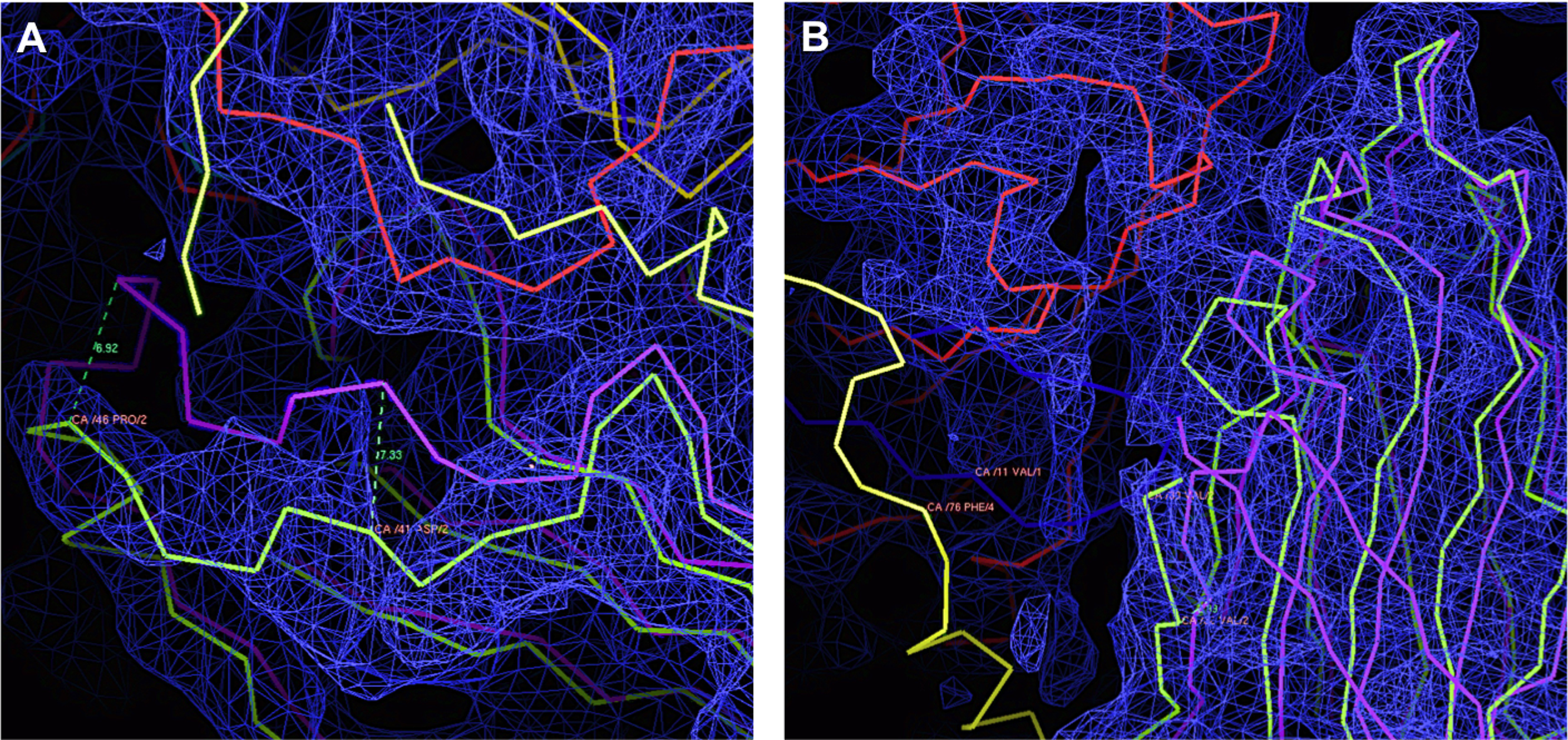

Supplement: S5 Fig — The angular sampling frequency (shown by the length of the rod) and distribution (shown by the rod direction) of particles of the isolated pentamer used to produce the 8.2 Å structure, drawn in Chimera. (TIF) [file ppat.1006607.s006.tif]

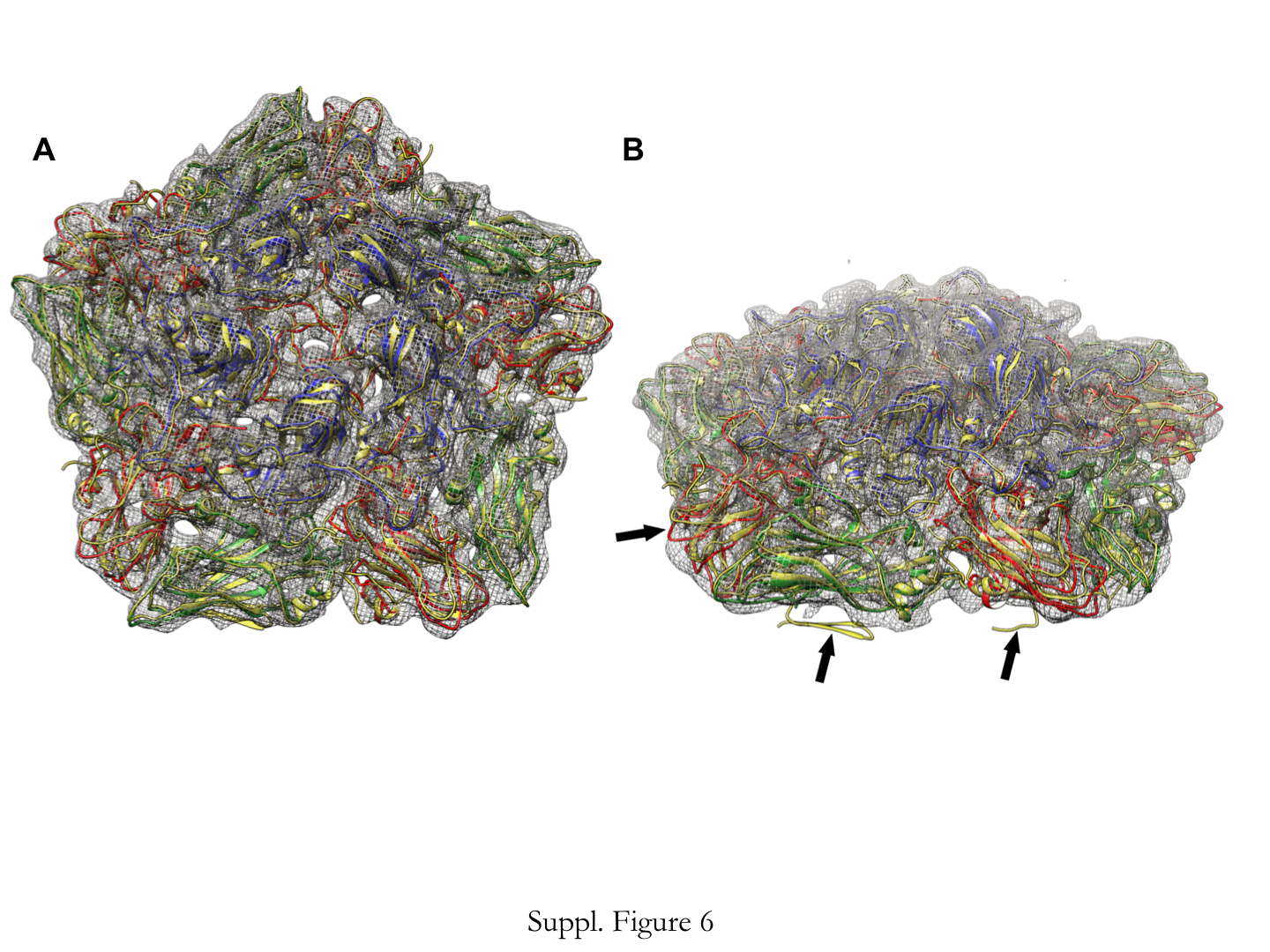

Supplement: S6 Fig — Two different views of the two pentamers shown to highlight the difference in the structures that allows the better fitting of the atomic model from the inside-out particle to fit into the electron density for the isolated pentamer. The view on the left is looking down on the pentamer, as if from outside the native virus, whilst the view to the right is almost edge-on with the outer surface in the native virion facing upwards. The missing density for the VP2 hairpin loop of the native structure and the better fitting of the VP3 (red) β-sheets from the inside-out particle model are visible (black arrows point to these structures). (TIF) [file ppat.1006607.s007.tif]

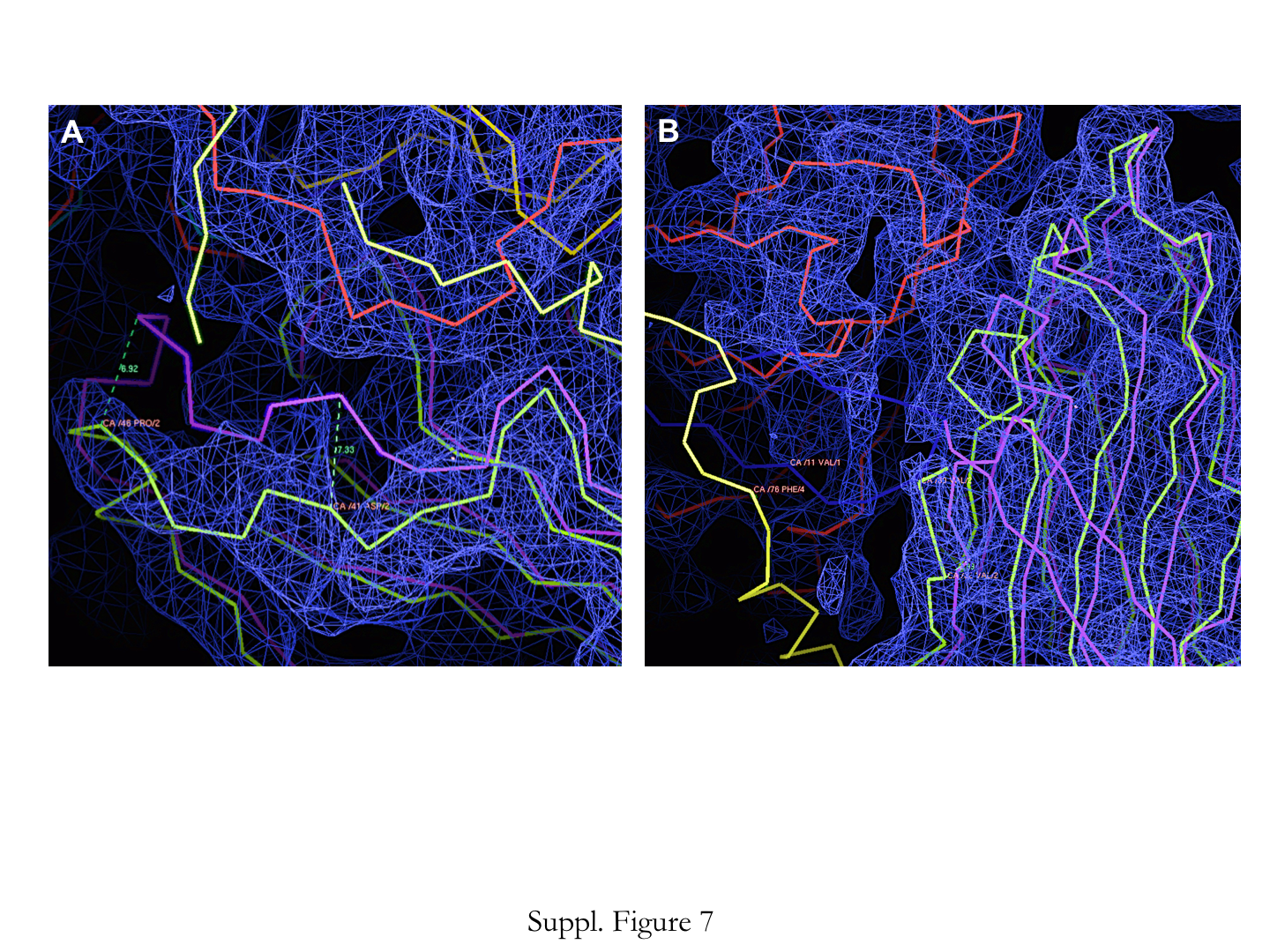

Supplement: S7 Fig — VP4 (yellow), the N-terminus of VP1 (dark blue in the background) in the case of the native capsid and VP3 (red) can also be seen. (TIFF) [file ppat.1006607.s008.tiff]
